# Polonium ^210^Po activities in human blood of patients with ischaemic heart disease from Gdańsk in Poland

**DOI:** 10.1007/s10967-013-2670-0

**Published:** 2013-08-02

**Authors:** Alicja Boryło, Bogdan Skwarzec, Grzegorz Romańczyk, Janusz Siebert

**Affiliations:** 1 Faculty of Chemistry, University of Gdańsk, Sobieskiego 18/19, 80-952 Gdańsk, Poland; 2 Department of Family Medicine, Medical University of Gdańsk, Skłodowskiej-Curie 3a, 80-210 Gdańsk, Poland

**Keywords:** Polonium, ^210^Po, Concentration, Human blood samples, Ischaemic heart disease, IHD, Cigarettes smoking, Fish consumption

## Abstract

The determination of polonium ^210^Po in human blood samples is presented and discussed in this paper. The human blood samples were collected from patients of Medical University of Gdańsk with ischaemic heart disease (*morbus ischaemicus cordis*, *MIC*). The polonium concentrations in analyzed human blood samples are very differentiated. ^210^Po is of particular interest in public health and although is present in the environment in extremely low amounts, it is easily bioaccumulated to the human body. The study shows that the amount of ^210^Po that is incorporated into the human body depends on the food habits and some difference in its levels could be observed between smokers and non-smokers.

## Introduction

Ischaemic or ischemic heart disease (IHD), or myocardial ischaemia, is a disease characterized by ischaemia (reduced blood supply) of the heart muscle, usually due to coronary artery disease (atherosclerosis of the coronary arteries). Its risk increases with age, smoking, hypercholesterolaemia (high cholesterol levels), diabetes, and hypertension (high blood pressure), and is more common in men and those who have close relatives with ischaemic heart disease. It is the most common cause of death in most industrialized countries, and a major cause of hospital admissions [1]. There is limited evidence for population screening, but prevention (with a healthy diet and sometimes medication for diabetes, cholesterol and high blood pressure) is used both to prevent IHD and to decrease the risk of complications.

Blood is a specialized bodily fluid in animals that delivers necessary substances such as nutrients and oxygen to the cells and transports metabolic waste products away from those same cells. In vertebrates, it is composed of blood cells suspended in a liquid called blood plasma. Plasma, which constitutes 55 % of blood fluid, is mostly water (92 % by volume), and contains dissipated proteins, glucose, mineral ions, hormones, carbon dioxide (plasma being the main medium for excretory product transportation), and blood cells themselves. Albumin is the main protein in plasma, and it functions to regulate the colloidal osmotic pressure of blood. The blood cells are mainly red blood cells (also called RBCs or erythrocytes) and white blood cells, including leukocytes and platelets. The most abundant cells in vertebrate blood are red blood cells. These contain hemoglobin, an iron-containing protein, which facilitates transportation of oxygen by reversibly binding to this respiratory gas and greatly increasing its solubility in blood. In contrast, carbon dioxide is almost entirely transported extracellularly dissolved in plasma as bicarbonate ion [1].

The natural radionuclide ^210^Po is daughter of ^238^U decay series. ^210^Po is radionuclide with half-lives of 138.38 days. Polonium is one of the most radiotoxic natural radioactive isotopes to man due to its high specific activity and its emission of high-LET alpha radiation. Less than 0.05 μg of the radionuclide is considered a lethal dose (LD_50/30_). This isotope was used to kill the Russian agent Andrei Litvinenko in 2006, by putting about 10 μg of ^210^Po in his tea [2, 3]. The ^210^Po is found in varying concentration in soil, sand, sediment and naturally occurring water. This radionuclide constitutes an important component of the natural background radiation and contributes significantly to the radiation dose of the population [4]. The main source of ^210^Po in the atmosphere is ^222^Rn emanation from the ground. ^210^Po returns to the earth as dry fallout or is washed out in rain. Other sources include burning of fossil fuels and tetraethyl lead in petrol, superphosphate fertilizers, the sintering of ores in steelworks, the burning of coal in coal-fired power plants [5, 6]. ^210^Po is highly toxic and its presence in soils may be traced to the decay of radionuclides of the ^238^U chain in the soil [7]. Man is exposed to radioactive ^210^Po by natural processes, mainly from the oral intake of foodstuff. Especially large amounts of polonium are taken in during cigarette smoking as well as food of marine products [8–12]. Most of the polonium entering the body orally reaches the gastrointestinal (GI) tract and is eliminated via excreta. The estimated contribution made by ^210^Po to the total annual background effective dose is 120 μSv [13]; which is about 5 % of the total estimated average global background to humans [3].

The aim of this study was to establish the polonium ^210^Po concentrations in blood samples. The tested group constituted patients from Medical University of Gdańsk. Ischaemic heart disease patients do not constitute a high-risk group as far as the concentration of ^210^Po in blood is concerned. However, in the treatment of this disease is recommended a diet rich in fish. Eating fish is a factor that according to many researchers affects the amount of this radionuclide in the human body. In many cases the reason of IHD is cigarette smoking. The questions about smoking and frequency of fish eating were included in our questionnaire for the patients. Thus, the content of ^210^Po in the body of the patients were examined and linked to the above mentioned factors. This is very important because human biomonitoring of ^210^Po has been conducted for a long time, but it is still not fully known and understood.

## Materials and methods

The human blood samples about volume 10 ml were collected from 43 patients (8 women and 35 men) with ischaemic heart disease, IHD (*morbus ischaemicus cordis*, *MIC*) from Medical University of Gdańsk. The reason for choosing this particular group was purely accidental. Age of patients ranged from 45 to 78 years, body weight between 55 and 110 kg, and the height of 155 to 185 cm. Research conduted by the Medical University of Gdańsk were part of a program entitled “The role of cytokines in the inflammatory process caused by coronary heart disease causing agents in patients with ischaemic heart disease”. The research was approved by the Independent Bioethics Committee for Scientific Research of the Medical University of Gdańsk.

Before radiochemical analysis, to each sample was added about 8 mBq of ^209^Po as yield tracer. The human blood samples were mineralized using of concentrated acids HNO_3_. After evaporation, the dry residue was dissolved in 10 ml 0.5 M HCl and, after the addition of ascorbic acid to reduce Fe^3+^, the solution was transffered to Teflon (PTEE) vessels equipped with a silver sheet bottom. Polonium was autodeposited at 90 °C for 4 h [14–16]. The activities of ^210^Po were measured using alpha spectrometer (Alpha Analyst S470) equipped in a surface barrier PIPS detector with an active surface of 300–450 mm^2^ placed in a vacuum chamber connected to a 1,024 multichannel analyzer (Canberra-Packard, USA). Detectors higher counting efficiency ranged from 0.30 to 0.40. Minimum Detectable Activity (MDA) measurement of ^210^Po was calculated as 0.1 mBq. Polonium preparates were measured for 2 days and ^210^Po activity was calculated at the time of its electrodeposition on silver discs. Time between collection blood samples and their radiochemical analysis was between 23 and 126 days. The polonium recoveries in analyzed samples ranged between 58 and 98 %. The results of ^210^Po concentrations in analyzed samples are given with standard deviation (SD) calculated for a 95 % confidence interval (±2σ). The concentrations of ^210^Po in the IAEA-300, IAEA-327, 384, 385 and IAEA-326, 414 samples were consistent with the reference values reported by the IAEA. The accuracy of the analytical method and measure of precision was estimated to be below 2.4 and 3 %, respectively. The content of ^210^Po activities in the total volume of blood in the patients has estimated on the basis [17]:

$$ {\text{for men}}:{\text{V}} = 0. 3 6 6 9\times {\text{G}}^{ 3} + 0.0 3 2 1 9\times {\text{W}} + 0. 60 4 1 $$

$$ {\text{for women}}:{\text{V}} = 0. 3 5 6 1\times {\text{G}}^{ 3} + 0.0 3 30 8\times {\text{W}} + 0. 1 8 3 3 $$

where G is the growth (m), W is the weight (kg), V is the total volume of blood

## Results

**Fig. 1:**
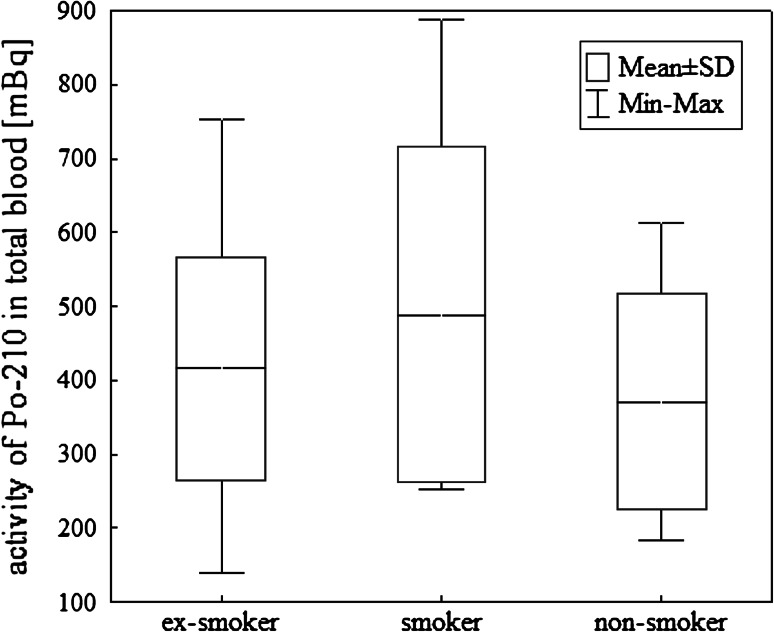
^210^Po activity in total blood in smokers, non-smokers and ex-smokers

**Fig. 2:**
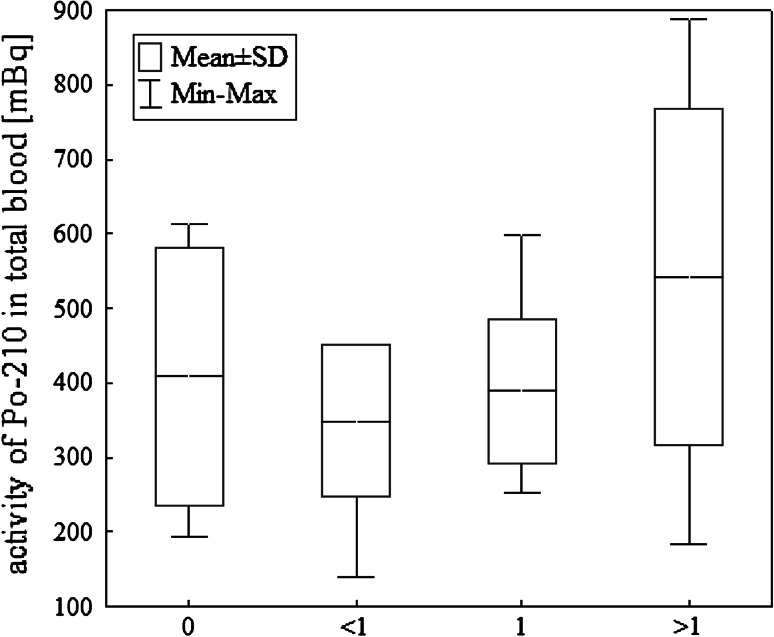
^210^Po activity in total blood of weekly fish consumption

**Fig. 3:**
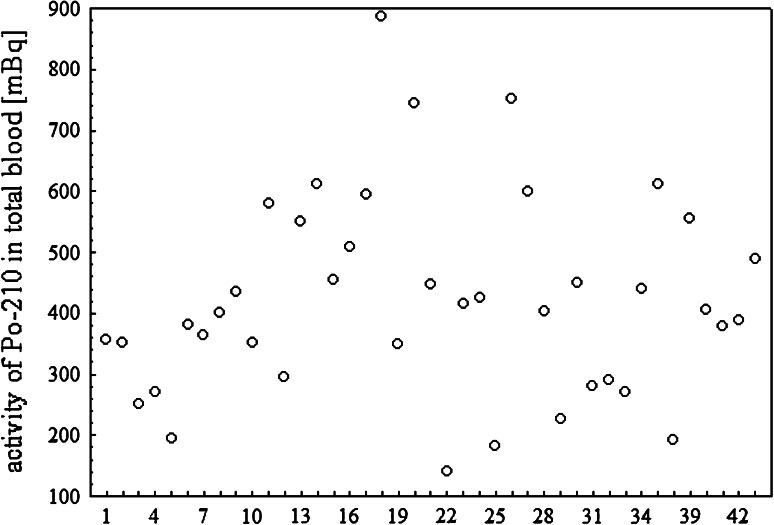
^210^Po activity in total blood for analyzed patients

The results of ^210^Po concentrations in analyzed human blood samples are presented in Table 1. ^210^Po concentration in the analyzed samples ranged between 32 ± 3 mBq dm^−3^ and 558 ± 47 mBq dm^−3^. In the total blood volume of analyzed patients the content of ^210^Po lies between wide range from 140 ± 14 mBq to 3,072 ± 270 mBq (Table 1). Two values of the obtained results indicate the maximum concentration of the analyzed ^210^Po (495 ± 44 mBq·dm^−3^ and 558 ± 47 mBq dm^−3^ or 3,072 ± 270 mBq and 2,901 ± 245 mBq in total blood). After their rejection the values of ^210^Po in analyzed samples lie between 140 ± 14 mBq in total blood and 888 ± 36 mBq in total blood. The higher ^210^Po activity was observed for males (33 samples), the lower for females (8 samples) (435 ± 36 mBq in total blood and 366 ± 33 mBq in total blood respectively). The results of ^210^Po activity in blood of smokers, non-smokers and ex-smokers groups are presented in Fig. 1. The results of ^210^Po activity of weekly fish consumption groups are given in Fig. 2 and results of this radionuclide content in total blood for all analyzed patients are given in Fig. 3. In the group of non-smokers (4 samples) the mean value ^210^Po activity was 362 ± 36 mBq in total blood. The higher values of ^210^Po activity were observed in groups of smokers and ex-smokers (6 and 31 samples respectively) (422 ± 34 mBq in total blood and 429 ± 35 mBq in total blood respectively). Our obtained results of ^210^Po activities in human blood are higher than results from Arabia, where the activity of ^210^Po ranged from 0.91 to 4.56 pCi/l (from 33.67 mBq dm^−3^ to 168.72 mBq dm^−3^) in blood of smokers with an average value of 1.83 ± 0.63 pCi/l (67.71 ± 0.63 mBq dm^−3^). Blood samples of non-smoker showed ^210^Po activity ranging from 0.61 ± 3.14 pCi/l (22.57–116.18 mBq dm^−3^) with an average value of 1.29 ± 0.61 pCi/l (47.73 ± 0.61 mBq dm^−3^) [18]. The mean value of smoker is significantly higher (about 30 %) than in non-smokers. The ^210^Po concentration in blood samples of human is very differentiated and some of the values, especially those which have been obtained for two patients (numbered 37 and 38) (3,072 ± 270 mBq and 2,901 ± 245 mBq in total blood) (Table 1) are difficult to explain. These difficulties arise from the lack of complete characterization of these people. Among the patients the interview was carried out about sex, frequency of cigarettes smoking and fish consumption. There are no data about the person with the number 37, except that it is a man. As far as the person with the No. 38 is concerned, it is only that he is a man, an ex-smoker who quite often eats fish. It is not known how long the patients suffer. These extremely high levels of polonium can be related to improper sampling, like diet of patients, the time between sampling of blood samples and their radiochemical analysis or depend on other factors. The majority of the samples were obtained from Medical University of Gdańsk within 6 months. No information was available concerning, for example their all feeding habits, nutritional supplements prior to blood sampling day, place of residence (rural or city), method sampling of blood and sexual activity, too. It is very significant because according to the literature about 300 % increase of ^210^Po concentration in blood was observed the day following consumption of fish and seafood in human semen fluid of vasectomized non-smoker volunteers. The level of polonium returned to near baseline after 4 days with a controlled diet, excluding fish and seafood [19].

**Table 1:** The ^210^Po concentration in human blood samples

Sample	^210^Po	
	Concentration	In total blood
	mBq dm^−3^	mBq
1	63 ± 6	358 ± 34
2	65 ± 5	353 ± 25
3	57 ± 7	252 ± 30
4	51 ± 5	270 ± 25
5	57 ± 4	195 ± 15
6	99 ± 9	381 ± 33
7	67 ± 7	363 ± 36
8	78 ± 7	402 ± 38
9	84 ± 8	435 ± 41
10	71 ± 7	352 ± 36
11	132 ± 12	579 ± 51
12	50 ± 4	295 ± 26
13	98 ± 7	551 ± 42
14	153 ± 15	612 ± 61
15	88 ± 10	455 ± 23
16	100 ± 13	508 ± 65
17	130 ± 11	594 ± 50
18	184 ± 18	888 ± 36
19	65 ± 7	350 ± 35
20	159 ± 15	744 ± 71
21	83 ± 7	447 ± 40
22	32 ± 3	140 ± 14
23	90 ± 8	416 ± 37
24	88 ± 7	425 ± 22
25	51 ± 5	183 ± 18
26	162 ± 10	753 ± 48
27	125 ± 6	599 ± 30
28	69 ± 5	404 ± 30
29	54 ± 6	226 ± 23
30	92 ± 9	450 ± 42
31	62 ± 5	281 ± 22
32	60 ± 4	291 ± 20
33	62 ± 7	271 ± 33
34	78 ± 7	440 ± 42
35	114 ± 10	612 ± 53
36	43 ± 5	193 ± 24
37	558 ± 47	2,901 ± 245
38	495 ± 44	3,032 ± 270
39	90 ± 7	555 ± 42
40	74 ± 7	405 ± 37
41	78 ± 7	378 ± 33
42	83 ± 7	389 ± 31
43	102 ± 8	488 ± 40

**Fig. 4:**
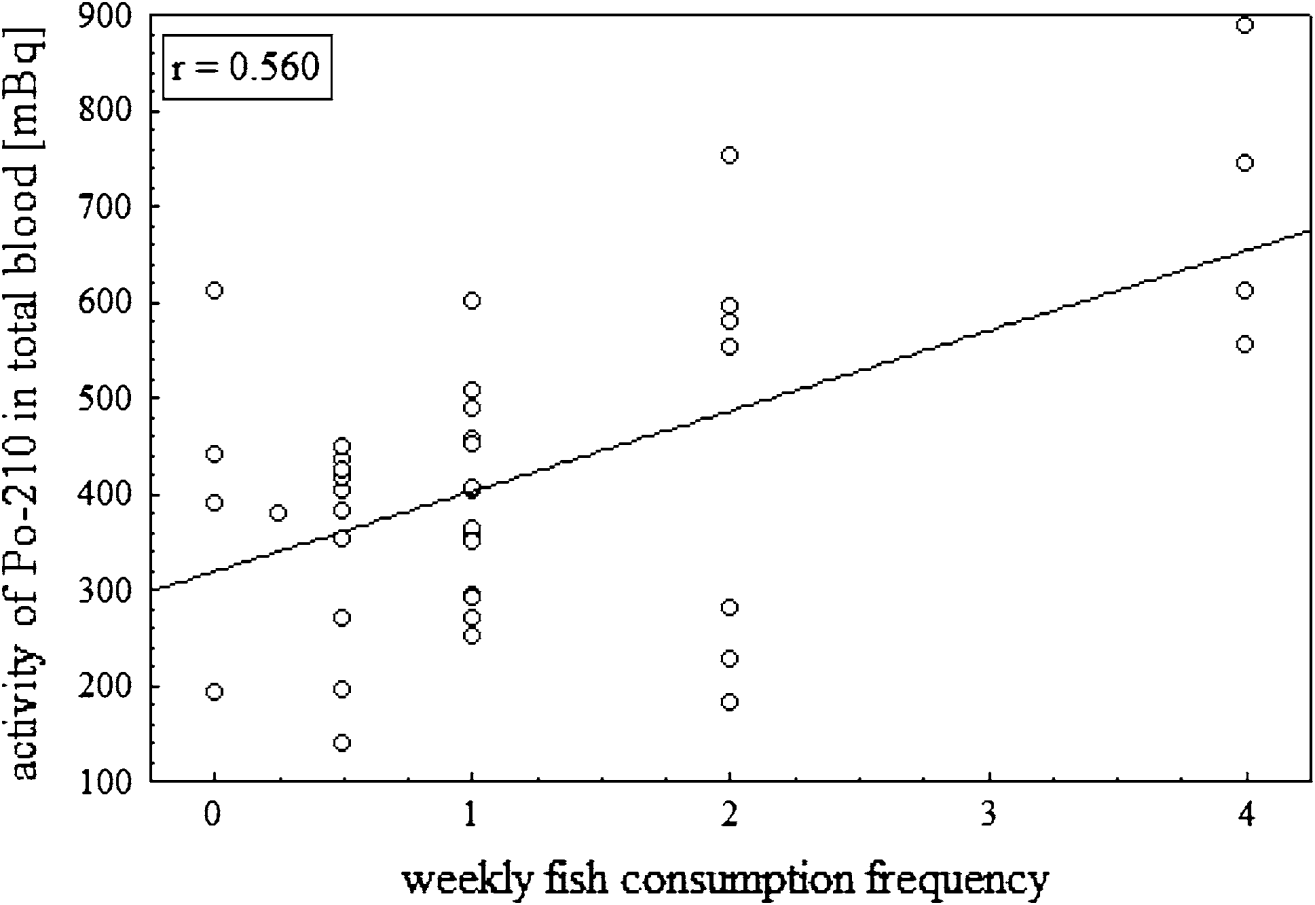
The correlation between ^210^Po activity in blood samples and patients habits

The reason for the high accumulation of polonium in the body is its affinity to protein, allowing it to pass easily through the food chain [3]. Despite that knowledge on the metabolic behavior of ^210^Po in humans is relatively scarce, but the activity of ^210^Po in different human tissues is following order: hair > bone > liver = kidney > gonads > spleen = lung > muscle > heart = blood [20]. The main source of ^210^Po intake by the human body is the ingestion with foodstuffs and drinking water. Other studies reported that cigarette smoking also represents a significant source [8, 21, 22]. The absorption coefficient of ^210^Po into blood from the digestive tract is estimated at 35 or 40 % [22–24]. The large amounts of polonium are observed in protein-rich food, such as shellfish and crustaceans, and also observed among populations consuming large amount of reindeer and caribou meat, e. g. in Subarctic area [25, 26]. Although, as pointed out Al-Masri and collaborations higher ^210^Po concentrations are found in the edible tissue of sea fish than in fresh water fish [27]. Figure 4 presents the correlation between the ^210^Po concentration in blood samples and patients habits. A relatively good correlation was obtained for a group of people who eat fish (Pearson correlation factor *r* = 0.560). The majority of analyzed patients in the age group between 60 and 75 years. They resident generally the Tricity areas and according with their habit, they buy sea fish from the area of the Gulf of Gdańsk for consumption [8, 12].

**Fig. 5:**
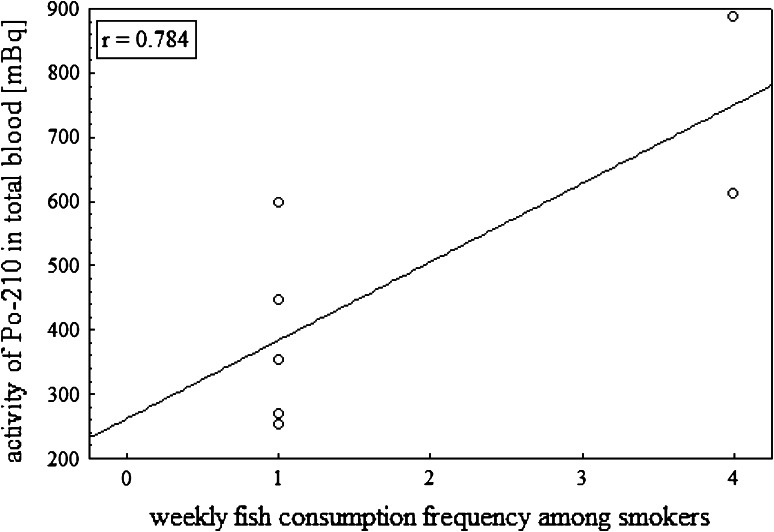
The correlation between ^210^Po activity in total blood of smokers group and consumption of fish

**Fig. 6:**
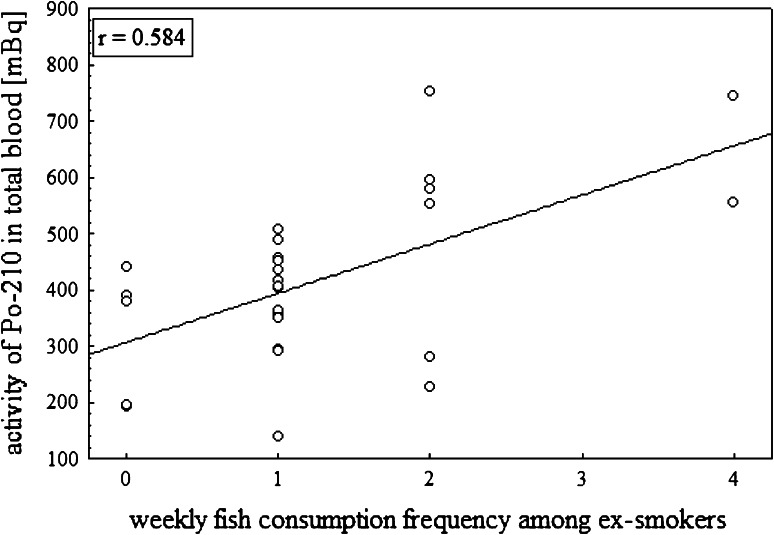
The correlation between ^210^Po activity in total blood of ex-smokers group and consumption of fish

The relatively high ^210^Po activity concentrations are found in tobacco and its products, well cigarette smoking highly increases the internal intake of this radionuclide and its concentrations in the lung tissues [9, 28]. The patients were subdivided into three classes and in every group cigarette smokers, non-smokers ad ex-smokers were taken into account. ^210^Po concentration in blood depends on the amount of cigarettes smoked per day and consumption of fish (Fig. 5). Our results show, that the cigarette smoking increases the content of ^210^Po in blood (Pearson correlation factor *r* = 0.784), but it should be noted that the analyzed group was less representative because of the number (only 7 of the 43 people). However, the group of smokers and ex-smokers combined together equals 36 people and as such constitutes a group which can considered representative. This group have higher ^210^Po concentration in blood than the group of non-smokers. Also Al-Arifi et al. [29] suggested that smoking is one significant route among other different routes of ^210^Po intake by human body and this effect was observed for more numerous smokers group in Saudi Arabia (51 persons who smoke and 23 persons who don’t smoke). Our studies are in accordance with other sources, where ^210^Po is invariably cited among the dangerous components of cigarette smoke [30], and responsible for at least 4 cases of cancer among 10,000 smokers [19, 31]. The difference between ^210^Po activities in human blood of ex-smokers and food habits is statistically significant (Pearson correlation factor *r* = 0.584) (Fig. 6). This obtained correlation is similar to correlation for a group of people who eat fish, which can explain the large abundance (33 of the 43 people).

There wasn’t any significant difference between the ^210^Po concentration and the age of patients and between males and females. Also in Turkey the difference was not statistically significant for lead between males and females [32]. The similar effect was observed for lead between girls and boys, but the levels of lead decreased significantly with age. The authors showed, that blood lead was associated with environmental noise and family income [33]. In our study there were no differences of age (about 85 % of the patients ranged in age from 60 to 70 years), thus probably has not been found significant statistical correlation.

Some differences between ^210^Po activities in human blood are connected with the time of blood sampling. The higher polonium activities were estimated in blood samples collected in the summer (554 ± 37 mBq in total blood), the smaller in blood samples collected in the winter (329 ± 25 mBq in total blood). ^210^Po is very broadly distributed in all environment. As already mentioned sources of ^210^Po are burning of fossil fuels and tetraethyl lead in petrol, superphosphate fertilizers, the sintering of ores in steelworks, the burning of coal in coal-fired power plants and phosphogypsum stockpiles [5, 6, 34]. Most of these processes are more intense in the summer. This fact may explain the higher polonium activities in blood samples in summer season.

Cigarette smoking raises blood pressure, probably through the nicotine-induced release of norepinephrine from adrenergic nerve endings, but not fully understood is the behavior in patients with ischaemic heart disease. In subjects with normal resting blood pressure and fixed myocardial perfusion defect (scar), cigarette smoking had no effect on exercise blood pressure [35]. The concentrations of ^210^Po in blood will probably depend here on the severity of the disease and its duration. It should also be noted that the content of trace metals and radionuclides in blood may vary depending on a variety of other factors. The studies conducted in India showed that the mean blood level for lead in stray dogs either from urban or rural locality was significantly higher than that of pets, and the blood lead concentration was significantly higher in nondescript dogs than pedigreed dogs. The locality (urban/rural) was the major variable affecting blood lead concentration in dogs. Breed and housing of the dogs of urban areas and only housing (pet/stray) in rural areas significantly influenced the blood lead concentration in dogs [26]. The other research showed that the specific activities of ^210^Po accumulated in tissues depend on the initial its contents radionuclide in animals’ food, too [36]. It allows to draw conclusion that similar effect on the human organism may be expected. Also the three times higher ^210^Po activities are observed in the area with a High Level Natural Radiation Area (Iran) [24] and various activities depend also on the place of residence of persons from whom blood samples were taken [37]. The similar effect was observed for the uranium concentrations in Iraq. The uranium concentrations in the blood samples of workers were found to increase with the increasing number of working years, and were higher than those of non-occupied persons in the different governorates of Iraq. The highest uranium concentration in the blood for non-occupied workers was found in Basrah and Al-Muthana governorates. The authors suggest that these two governorates were the centers of intensive military activities during the 1991 war, and the discarded weapons are still lying around in this region [38]. This problem is discussed in the world [39], where many researchers are looking for methods to apply for multi-element determination of trace elements in whole blood as well as in human hair. This is very important to establish for further research for occupationally-exposed population or population under possible risk, such as workers in industrial plants [40].

Smoking is one of the three most powerful and potential risk factors for ischaemic heart disease. Smoking of cigarettes nearly doubles disease risk and increases three times the risk of sudden death. The risk increases with the increasing number of smoked cigarettes per day and decreases after finishing smoking. Although mortality decreased after 5 years of the end of habit, it was still higher than in non-smokers. Eating oily fish twice a week may help prevent heart attacks. This is connected with the properties of fish oil fatty acid, which prevents the excessive thickness of the vascular wall and thus improves the conditions of the blood supply to the heart. ^210^Po is accumulated by fish, whose consumption reduces the risk of ischaemic heart disease. This does not mean that the main source of polonium in human is of this origin. The content of polonium was higher in the analyzed blood samples of smokers and ex-smokers in comparison with non-smokers. It seems, therefore, that the values of ^210^Po activities in human blood of patients with ischaemic heart disease are only a result of smoking. Metarion et al. also found no significant changes in the concentrations of analyzed elements (Ca, Cl, K, Mg) in the blood of patients with chronic kidney disease when compared with healthy individuals and suggest that any changes could be related to nutritional habits, medicine ingestion as well as the evolution of the CKD [41]. Also the results of research in Iran revealed that the difference between the concentration levels of Br, Fe, and Zn in samples from patients affected by multiple sclerosis and control group was not meaningful. The average level of Zn in two analyzed samples is significantly different which suggests that the shortage of Zn can be one of the causes of MS, but the authors suggested that results of measuring the level of Zn in patients’ blood by other investigators are contradictory [42].

## Conclusions

The results of this work indicate that the activity of ^210^Po in human blood was in the wide range between 140 ± 14 mBq and 888 ± 36 mBq in total blood without two patients (3,072 ± 270 mBq and 2,901 ± 245 mBq in total blood). The higher activity of this radionuclide was observed for smoker and ex-smoker groups. The difference between ^210^Po activities in human blood of ex-smokers/smokers and eating habits is statistically significant. The patients were subdivided in groups: males and females, cigarette smokers, non-smokers and ex-smokers were taken into account. The results indicated that the ^210^Po activity was widely distributed in the each group of analyzed patients. The obtained results of ^210^Po activity in the blood of patients with ischaemic heart disease are probably related to the consumption of fish and smoking. The polonium activities in blood are not connected with degree of disease advancement.
